# Rotating Directional Solidification of Ternary Eutectic Microstructures in Bi-In-Sn: A Phase-Field Study

**DOI:** 10.3390/ma15031160

**Published:** 2022-02-02

**Authors:** Kaveh Dargahi Noubary, Michael Kellner, Britta Nestler

**Affiliations:** 1 Institute for Applied Materials-Computational Materials Science (IAM-CMS), Karlsruhe Institute of Technology (KIT), Straße am Forum 7, 76131 Karlsruhe, Germany; michael.kellner@kit.edu (M.K.); britta.nestler@kit.edu (B.N.); 2 Institute of Digital Materials Science (IDM), Karlsruhe University of Applied Sciences, Willy-Andreas-Allee 19, 76131 Karlsruhe, Germany

**Keywords:** rotating directional solidification (RDS), phase-filed simulation, anisotropic interfacial energies, tilted lamellar growth, ternary eutectics, Bi-In-Sn

## Abstract

For the first time, the experimental processing condition of a rotating directional solidification is simulated in this work, by means of a grand-potential-based phase-field model. To simulate the rotating directional solidification, a new simulation setup with a rotating temperature field is introduced. The newly developed configuration can be beneficent for a more precise study of the ongoing adjustment mechanisms during temperature gradient controlled solidification processes. Ad hoc, the solidification of the ternary eutectic system Bi-In-Sn with three distinct solid phases α,β,δ is studied in this paper. For this system, accurate in situ observations of both directional and rotating directional solidification experiments exist, which makes the system favorable for the investigation. The two-dimensional simulation studies are performed for both solidification processes, considering the reported 2D patterns in the steady state growth of the bulk samples. The desired αβαδ phase ordering repeat unit is obtained within both simulation types. By considering anisotropy of the interfacial energies, experimentally reported tilted lamellae with respect to normal vectors of the solidification front, as well as predominant role of αβ anisotropy in tilting phenomenon, are observed. The results are validated by using the Jackson–Hunt analysis and by comparing with the existing experimental data. The convincing agreements indicate the applicability of the introduced method.

## 1. Introduction

In recent years, the phase-field method has become a powerful and versatile technique to study microstructure evolutions in different material systems within simulations [1,2,3]. By this technique, various patterns, evolved in the solidification processes, such as dendrites [4,5,6], eutectics [7,8,9], and peritectics [10,11] have been investigated. A general introduction to this method is given in [12]. Focusing on the eutectics, in all mentioned simulation studies, the *directional solidification (DS)* processes have been investigated by using a Bridgman furnace setup. In this setup, the involved solid phases grow into the liquid phase, guided by the applied temperature field. Depending on the system and the process parameters, different growth morphologies and instabilities, such as tilted growth [13,14,15,16] and oscillations in the solid phases boundaries [17,18], have also been reported in the literature. Following the theory of Jackson and Hunt [19], for a constant velocity of the applied temperature gradient and benefiting domain sizes, a straight lamellar growth of isotropic solid phases is expected in two-dimensional simulations.

Next to the directional solidification process, a further experimental setup, called *rotating directional solidification (RDS)*, has been introduced by Oswald et al. [20] and Akamatsu et al. [21], in order to study the solidification of eutectic structures in thin samples. This rotation leads to the curved trajectories of the solidified phases and to a variation of the growth velocities at the solidification front. In these experimental setups, the sample is rotated with respect to an applied thermal gradient field with a predefined angular velocity. Hence, the growth velocities of the solids for example, vary proportionally to the distance from the center of rotation [20,21,22].

Accordingly, due to the significant effect of the solidification velocity on the pattern formation [23,24,25,26], the existence of multiple velocities within a single experiment allows studying the interaction of neighboring lamellae with different growth velocities. This can give a better insight into the ongoing velocity-controlled mechanisms in the solidification process and is considered as one of the main advantages of the *RDS* technique to the *DS* process. A further advantage is the possibility to efficiently study the effects of anisotropic behaviors in the interfacial energies γ on the pattern formation. For grains with a negligible solid/solid interface anisotropy *(floating grains)*, circular growth trajectories have been observed in *RDS* experiments. On the contrary, a tilted growth of the solidified phases, with respect to the solid/liquid interface normals, occurs for grains with significant anisotropy amounts *(locked grains)* [21,22]. Rátkai et al. [27] have studied the effects of the interfacial energy anisotropies on the pattern formation in binary eutectic Ag-Cu system by means of the phase-field method. In order to mimic the process condition of the *RDS* experiments, they have considered an angle between the anisotropy function long axis and the solidification direction. As in their study no rotating disk nor rotating temperature field is modeled, the obtained microstructures have waved lamellar patterns tending to semi-circles by increase in the anisotropy strength (see Figure 5 of [27]). In addition, simulation studies with possibility of a full rotation can give new insights in role of the anisotropy in pattern formation, which have not so far been conducted in the literature.

Apart from the above mentioned advantages of the *RDS* technique, it is worth mentioning that the method is limited to thin sample observations or bulks of materials with 2D growth patterns in steady states. The ternary eutectic system Bi-In-Sn is an example of such a system with a 2D lamellar microstructure [28]. Moreover, due to its low melting point (≃332 K [29]), Bi-In-Sn has become a practical material system for an in situ observation of the ongoing mechanisms in solidification processes. In the works of [23,30,31,32], the evolved microstructures from *DS* experiments are reported for different growth rates and experimental conditions. Based on the results, three-phase lamellar patterns with αβαδ stacking sequences are obtained in the stable growth regions of the floating grains, in which α denotes the $BiIn_{2}$ phases, β denotes the β−In phases, and δ represents the γ−Sn crystalline phases, respectively. In their investigated 2D samples, Witusiewicz et al. [30] have shown that the resultant amounts of the lamellar spacings for different solidification velocities are in good agreement with the Jackson–Hunt relationship [19]. They also show this agreement for previously reported results in bulk samples by Ruggiero et al. [31]. Later, in the work of Bottin-Rousseau et al. [23], which includes a very restricted velocity range, it is shown that the Jackson–Hunt relationship constant ($c=v\lambda^{2}$) agrees well with the results of [30]. On the other hand, in case of locked grains, anisotropy can affect the microstructure, in which small domains of αβδ or ${\left[\alpha \beta \right]}_{a}{\left[\alpha \delta \right]}_{b}$ superstructure are observed, wherein *a* and *b* are higher than unity integers [23]. Mohagheghi et al. [22] have conducted an *RDS* study of the system Bi-In-Sn in thin samples and have observed the same αβαδ stacking sequences as found in *DS* experiments with circular trajectories. This structure is reported to be consistent for a wide variation range of the affecting parameters, such as the sample thickness and the growth velocity. However, a tilted growth of the solid phases has also been observed in the locked grains. It has been observed that the αβ interface anisotropy has a more affective role in the formation of the inclined lamellae compared with an anisotropy of the αδ interface [22]. This investigated behavior of the interfacial anisotropies is another reason which makes the system Bi-In-Sn favorable for the first investigations of the rotating directional solidification process within phase-field simulations.

In order to simulate the *RDS* process, the utilized phase-field model is first introduced and the temperature formulation, which is to resemble the effects of the rotating temperature field, is presented. Then, the thermodynamic modeling of the system is shown, using the method from Noubary et al. [33] and the Calphad database from Witusiewicz et al. [29]. Next, the simulation setup and the used parameters are introduced. To validate the generated material system, *DS* simulations are performed in the desired growth velocity ranges of the experimental *DS* and *RDS* studies [22,30], respectively. The mentioned validation is performed by Jackson–Hunt analysis and by comparing the obtained results with the reported experimental data in the literature. Finally, the phase-field studies of the rotating directional solidification are performed, resulting in the circular trajectories of the solidified phases for the floating grains and inclined trajectories with respect to the solid–liquid interface normals, for the locked grains. The obtained results are compared with the existing experimental data of [21,22,30] In a last step, the made observations are summarized and discussed and an outlook to upcoming simulation studies is given.

## 2. Methods

For the simulation studies in this work, the grand potential functional [34,35] serves as the basis of the utilized phase-field model. The model is explicitly described in [36,37,38], while its utilization in the simulation of the directional solidification process has been reported for example in [13,38,39]. The following descriptions of the phase-field model are implemented into the multi-physics phase-field-framework Pace3D, version 2.4 [40,41]. By considering *N* order parameters $\phi_{\hat{\alpha }}$ for the involving phases, the local phase fractions are stored in the vector ϕ. The phases are labeled by the Greek letters α,β,δ,⋯. To avoid a confusion with the parameter of the interfacial energies $\gamma_{\hat{\alpha }\hat{\beta }}$, the Greek letter γ is not used to describe phases. The indices of the phases are marked with hat symbols $\left(\hat{□}\right)$, in order to distinguish phase counters by a disparate labeling. The vector μ indicates the corresponding *K* amounts of the chemical potentials $\mu_{i}$. Based on an Allen–Cahn approach and Fick’s law, the time evolution equations $\frac{\partial }{\partial t}$ of the coupled phase fields and the chemical potentials are formulated as:

$$\begin{array}{cc} \tau \varepsilon \frac{\partial \;\phi_{\hat{\alpha }}}{\partial \;t\;}= & -\underset{:=rhs_{1,\hat{\alpha }}}{\underset{︸}{\varepsilon \left(\frac{\partial \;a(\phi ,\nabla \phi )}{\partial \;\phi_{\hat{\alpha }}\;}-\nabla \cdot \frac{\partial a(\phi ,\nabla \phi )}{\partial \nabla \phi_{\hat{\alpha }}}\right)-\frac{1}{\varepsilon }\frac{\partial \;w(\phi )}{\partial \;\phi_{\hat{\alpha }}\;}}} \end{array}$$

(1) $$\begin{array}{cc} \; & -\underset{:=rhs_{2,\hat{\alpha }}}{\underset{︸}{\frac{\partial \;\psi (\phi ,\mu ,T)}{\partial \;\phi_{\hat{\alpha }}\;}}}-\underset{:=\Lambda }{\underset{︸}{\frac{1}{N}\sum_{\hat{\beta }=1}^{N}\left(rhs_{1,\hat{\beta }}+rhs_{2,\hat{\beta }}\right)}}\;,\\ \frac{\partial \;\mu }{\partial \;t\;}= & {\left[\sum_{\hat{\alpha }=1}^{N}h_{\hat{\alpha }}\left(\phi \right)\left(\frac{\partial c_{\hat{\alpha }}\left(\mu ,T\right)}{\partial \mu }\right)\right]}^{-1}(\nabla \cdot \left(M\left(\phi ,\mu ,T\right)\nabla \mu -J_{at}\left(\phi ,\mu ,T\right)\right) \end{array}$$

(2) $$\begin{array}{cc} \; & -\sum_{\hat{\alpha }=1}^{N}c_{\hat{\alpha }}\left(\mu ,T\right)\frac{\partial h_{\hat{\alpha }}\left(\phi \right)}{\partial t}-\sum_{\hat{\alpha }=1}^{N}h_{\hat{\alpha }}\left(\phi \right)\left(\frac{\partial c_{\hat{\alpha }}\left(\mu ,T\right)}{\partial T}\right)\frac{\partial \;T}{\partial \;t\;}), \end{array}$$

In Equation (1), a diffuse interface is exploited to model the phase evolution in the simulation procedure. The gradient energy density a(ϕ,∇ϕ), the potential energy density w(ϕ), and the driving force ψ are the key parameters that define the shape of the interface [38]. In order to resemble effects of anisotropy in the interfacial energies, a cubic positive anisotropy as given in [42] is applied. In this formulation, the gradient energy density is expressed as

(3) $$\begin{array}{c} a\left(\phi ,\nabla \phi \right)=\sum_{\hat{\alpha }<\hat{\beta }}\gamma_{\hat{\alpha }\hat{\beta }}{\left(a_{c}\left(q_{\hat{\alpha }\hat{\beta }}\right)\right)}^{2}{\left|q_{\hat{\alpha }\hat{\beta }}\right|}^{2} \end{array}$$

with $\gamma_{\hat{\alpha }\hat{\beta }}$ as the interfacial energy parameter, $q_{\hat{\alpha }\hat{\beta }}=\phi_{\hat{\alpha }}\nabla \phi_{\hat{\beta }}-\phi_{\hat{\beta }}\nabla \phi_{\hat{\alpha }}$ as the generalized gradient vector and $a_{c}\left(q_{\hat{\alpha }\hat{\beta }}\right)$ as anisotropy function. The cubic positive anisotropy is defined as

(4) $$\begin{array}{cc} a_{c}\left(q_{\hat{\alpha }\hat{\beta }}\right) & =1-\zeta_{\hat{\alpha }\hat{\beta }}\left(3-4\frac{{\left|q_{\hat{\alpha }\hat{\beta }}\right|}_{4}^{4}}{{\left|q_{\hat{\alpha }\hat{\beta }}\right|}^{4}}\right) \end{array}$$

with $\zeta_{\hat{\alpha }\hat{\beta }}$ as its strength [42]. The potential energy density function is described in [38] and includes the interfacial energies $\gamma_{\hat{\alpha }\hat{\beta }}$ and the higher-order term $\gamma_{\hat{\alpha }\hat{\beta }\hat{\delta }}$. This higher order term is introduced, so as to suppress a third-phase appearance at the phase boundaries. $\gamma_{\hat{\alpha }\hat{\beta }\hat{\delta }}$ is adjusted to reflect the correct equilibrium angle conditions at the triple junctions [38,42]. The thickness of all interfaces is controlled by the parameter ε and its kinetics is described by the relaxation coefficient τ [35]. In Equation (1), the Lagrange multiplier Λ is exploited to accomplish the constraint $\sum_{\hat{\alpha }=1}^{N}\partial \phi_{\hat{\alpha }}\;/\;\partial t=0$. In Equation (2), the evolution of μ is considered to characterize the diffusion processes. The information of the diffusion coefficient matrix D for the involved phases is included in the function M(ϕ,μ,T) [35] as the mobility, and the function $h_{\hat{\alpha }}\left(\phi \right)$ [43] interpolates between the different phases. As is common in phase-field models, the widths of the interfaces are orders of magnitude larger than their physical values [34], while the anti-trapping current $J_{at}$ [44,45] helps to adjust the influences of these nonphysically enlarged interface widths. The concentrations of the *K* chemical elements in the involved phases, are saved in the vector $c_{\hat{\alpha }}\left(\mu ,T\right)$ including *K* components. The driving force is defined by the grand potential deviations Δψ of the evolved phases. $\psi_{\alpha }$, for example, describes the grand potential of phase α. All grand potentials depend on the phase-field vector, the chemical potentials and the temperature   *T* and are stored in the vector ψ(ϕ,μ,T). Together with the concentrations $c_{\alpha }\left(\mu ,T\right)$ and the chemical potentials μ, the grand potentials can be derived from Calphad databases, by using the general method introduced in [33]. In this method, the volume and the pressure are assumed to be constants, which ensures the thermodynamic consistency of the system. The numeric algorithm to solve the system of Equations (1) and (2), includes a spatial discretization scheme. Based on this scheme, the amounts of the phase-fields and chemical potentials at current time *t* are considered as the inputs at each grid cell of the simulation domain. Initially, the phase-fields at time t+dt are calculated from the inputs in which dt stands for the considered time step. The outcome is utilized to calculate the chemical potentials at t+dt. All these calculations are performed considering the amounts in the neighboring cells in forming the discretization scheme [36]. The spatial derivations in the coupled set of the partial differential equations is discretized with finite differences and their time evolution is solved by an explicit Euler scheme [46].

As the evolved microstructures of the bulk samples are experimentally reported to be 2D in the steady states [28], the investigated samples in this work are considered to be two-dimensional, and a 2D heat distribution is assumed in the temperature field formulation. However, the derived formulation can still be used in the 3D cases of such material systems. Considering the solution of the heat equation in steady-state $\nabla^{2}T=0$, the temperature formulation can be expressed as following in form of a linear function with *space* and *time* as its variables:

(5) $$\begin{array}{c} T\left(x,t\right)=T_{0}+G^{\mathrm{DS}}\left(x-vt\right). \end{array}$$

**Figure 1:**
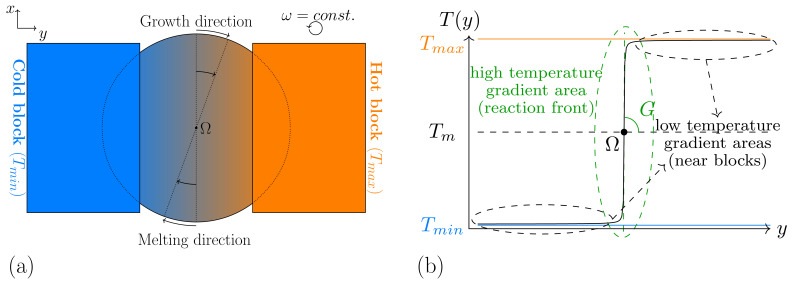
(**a**) Schematic illustration of the setup in the rotational solidification experiment, based on Mohagheghi et al. [22], (**b**) General form of the utilized temperature profile at time = 0, used in this paper. The areas with a high and low temperature gradient, respectively, indicate the solidification front and the areas with hot and cold blocks.

As utilized in the works of [13,47,48] for *DS* simulations, in this formulation $T_{0}$ indicates the base temperature, $G^{\mathrm{DS}}$ denotes the temperature gradient, *v* is the temperature gradient velocity, *t* represents the simulation time, and *x* refers to the growth direction, respectively. Hence, a linear temperature increase occurs in the solidification direction, with an overall decrease over time. In case of *RDS* simulations, despite rotating the sample as described in the depicted experimental setup in Figure 1a, a rotation of the temperature profile is realized within the Pace3D framework. By considering a rotating temperature field, the same physical effects can be reproduced with a reduced computational effort. In order to derive such a temperature formulation, moreover to satisfaction of the heat distribution equation, the following constraints should be considered:

(i) The sought formulation for the temperature has to represent the effects of the hot and cold isothermal blocks, the effects of the temperature profile in between, and the caused variations of the temperature, due to the rotation. To resemble the near uniform distribution of the heat in the vicinity of the blocks, a low temperature gradient is required in these segments, whereas in the segments nearer to the disk center, containing the solidification front, a sharper temperature change is necessary. Hence, a linear function for the whole simulation domain with a constant temperature gradient amount, as described in Equation (5), is not favored. By using such a function, the system temperature can rise dramatically with increasing distance from the rotation center, which can lead to a destabilization of the modeled material system, specially in large domain simulations.
(ii) To ensure a correct calculation of the evolution equations (Equations (1) and (2)), a continuously differentiable function is needed for the temperature, with respect to the space, in which differing amounts of the derivatives can exist in different space points. Based on this constraint, a piecewise function, composed of three linear functions with different temperature gradients can not be considered due to non-continuity of the first derivatives in the connection points.

One option to fulfill these constraints is the usage of a $tan^{-1}$ function, as schematically illustrated in Figure 1b. In this case, the upper and lower asymptotes of the function can resemble the hot and cold temperature blocks or the desired maximum and minimum temperatures in the simulation domain. The higher temperature gradient in the middle, physically represents the sharper temperature change at the disk center in comparison with the outer side. With this, well-defined solidification and melting fronts can establish themselves within the simulations. In order to formulate the discussed temperature profile, the start setting without disk rotation is considered first. By using the applied coordinate system of Figure 1a, the temperature is formulated as a function of *y*, in the form:

(6) $$\begin{array}{c} T\left(y\right)=T_{m}+A_{0}\cdot tan^{-1}\left(\frac{G^{\mathrm{RDS}}\cdot y}{A_{0}}\right), \end{array}$$

in which $T_{m}$ is the temperature and $G^{\mathrm{RDS}}$ is its gradient in the rotation center (x=0,y=0). $A_{0}$ is a constant coefficient to determine the amounts of the mentioned asymptotes. In this formulation, $\frac{dT}{dy}{|}_{y=0}$ will be equal to $G^{\mathrm{RDS}}$, as expected. Next, the rotational matrix is used to model the angle of rotation θ=ωt, depending on the time *t*, with an angular velocity of ω. The new coordinate system ($x^{\prime }-y^{\prime }$) is obtained as:

(7) $$\begin{array}{c} \left[\begin{array}{c} x^{\prime }\\ y^{\prime } \end{array}\right]=\left[\begin{array}{cc} cos(\omega t) & -sin(\omega t)\\ sin(\omega t) & cos(\omega t) \end{array}\right]\left[\begin{array}{c} x\\ y \end{array}\right]=\left[\begin{array}{c} x\cdot cos(\omega t)-y\cdot sin(\omega t)\\ x\cdot sin(\omega t)+y\cdot cos(\omega t) \end{array}\right]. \end{array}$$

The combination of Equations (6) and (7) results in the final space- and time-dependent formulation for the rotating temperature profile:

(8) $$\begin{array}{c} T\left(x,y,t\right)=T_{m}+A_{0}\cdot tan^{-1}\left[\frac{G^{\mathrm{RDS}}}{A_{0}}\left(x\cdot sin(\omega t)+y\cdot cos(\omega t)\right)\right]. \end{array}$$

The used parameters for $T_{m}$ and $A_{0}$ are given in Table A3 in the Appendix A. With these parameters and utilization of Equation (8), the average deviation form the exact solution of the steady-state heat equation $\nabla^{2}T$ in the simulation points is calculated as $2.5\cdot 10^{-25}$. This deviation is negligible in authors’ opinion. The introduced description of a rotating temperature field is used to perform the subsequently shown *RDS* phase-field simulation studies. It is worth mentioning that the implemented temperature formulation is independent from the investigated material system.

## 3. Simulations and Results

In this section, the modeling of the material system Bi-In-Sn is described for the simulation of the directional and the rotating directional solidification processes. The thermo-physical properties of the system are summarized in Table A4 of Appendix A. Additionally, the used simulation setups are introduced for both cases and consequently obtained results are presented.

### 3.1. Modeling the System Bi-In-Sn

To ensure a thermodynamically consistent modeling of the system Bi-In-Sn, the driving forces for the phase transitions within the phase-field simulations are calculated based on the thermodynamic Gibbs energies, stored in the Calphad database, taken from Witusiewicz et al. [29]. In the solidification process, a new computationally efficient formulation of the Gibbs energies is generated for all involved phases, on the basis of the stored information. From these new Gibbs energy formulations, other thermodynamic properties, such as the chemical potentials and the grand potentials, can be calculated [49]. In the course of this endeavor, the general procedure introduced in [33], for the approximation of the Gibbs energies of the ternary eutectic material systems, is used in this work. This approximation procedure contains the following five essential steps: In the first step, the equilibrium concentrations for the given working temperatures below the melting point are determined for all involved phases, using the Calphad databases. This is completed by using the commercial tool Thermo-Calc [50]. Next, the Gibbs energies for these phases are calculated in the vicinity of the equilibrium concentrations for the investigated temperatures. In the next step, new Gibbs energy formulations are approximated independently for each phase and temperature, based on the calculated values, using second-order polynomials. The use of second-order polynomials for the approximation of free energies is also suggested by Yang et al. [51]. Subsequently, the generated Gibbs energies are interpolated to model temperature-dependent functions. In the last step, the resultant functions are validated by measuring the maximum and average deviations between the Calphad data and the approximated data, as well as by rebuilding the equilibrium conditions of the phases. It has to be mentioned that the used method of [33] is limited to binary and ternary systems without stoichiometric phases. As the observed system Bi-In-Sn fulfills these conditions, this method is selected for the modeling. An alternative approach for the modeling of stoichiometric phase is introduced, for example, in [52].

**Figure 2:**
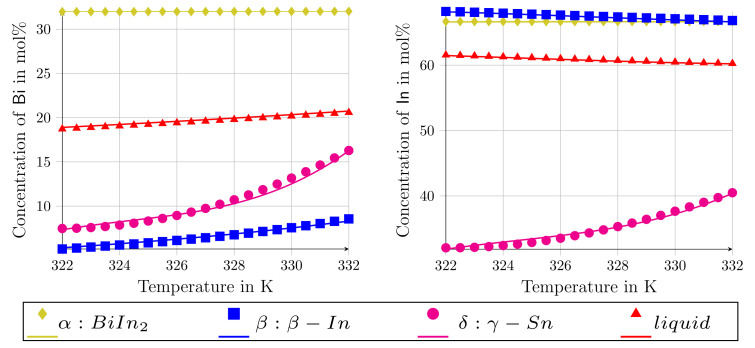
Comparison of the equilibrium concentrations of the evolving phases, occurring during the eutectic solidification of the Bi-In-Sn ternary system, with the Calphad database. In (**left**) the concentrations of Bi, and in (**right**) In concentrations are depicted. The solid lines denote the approximated Gibbs energy functions in this work, while the points refer to the Calphad database of [29].

For a working temperature of $T_{w}=331\;$ K, taken from the Calphad database [29], Table 1 exemplarily shows the results of the equilibrium calculations in step one. The calculated equilibrium concentrations are in good correlation with the experimentally reported near eutectic compositions in [28,30]. For the approximation of the Gibbs energies, second-order polynomials of the form $G\left(c_{\mathrm{Bi}},c_{\mathrm{In}},T\right)=a_{0}\left(T\right)\;c_{\mathrm{Bi}}{}^{2}+a_{1}\left(T\right)\;c_{\mathrm{In}}{}^{2}+a_{2}\left(T\right)\;c_{\mathrm{Bi}}\;c_{\mathrm{In}}+a_{3}\left(T\right)\;c_{\mathrm{Bi}}+a_{4}\left(T\right)\;c_{\mathrm{In}}+a_{5}\left(T\right)$ are used. The generated parameters, $a_{i}$ with i=0,1,...,5, for the phases $BiIn_{2}$, β−In, γ−Sn and liquid, are summarized in Table A1 of Appendix A. To validate the approximated functions, the derived values of the Gibbs energies and the chemical potentials from these functions are listed in Table A2, for the exemplary temperature of 331 K, together with the calculated values from the Calphad database for this temperature. For all phases, the maximum deviation between the derived and calculated Gibbs energy amounts is less than 0.1%, while the maximum deviation between the chemical potentials is less than 2%. In Figure 2, the equilibrium conditions of the involved phases at different temperatures are directly compared with the calculated equilibrium states of the Calphad data, so as to test the validity of the approximated functions for a larger temperature range. As can be seen, the formulations represent the equilibrium concentrations of the elements (including the solubility shifts) for a large range of the temperature variation beneath the eutectic reaction. These observations demonstrate a good agreement between the Calphad data and the approximated functions, making good prerequisites of simulation studies in the upcoming sections.

### 3.2. Simulation Setup and Parameters

To validate the resulting microstructures of the subsequently performed simulations for the rotating directional solidification, directional solidification simulations are executed in advance. The utilized setup for the *DS* simulations, for example, is described in the works [13,48]. In this configuration, the initial filling of the phases consists of the solid phases with the desired αβαδ arrangement beneath the liquid phase. In order to resemble the infinite liquid flux in the simulation, a Dirichlet boundary condition is imposed to the ultimate liquid side. The opposite side, including the initial filling of the solid phases, is modeled by a Neumann boundary condition. Periodic boundary conditions are applied in the directions perpendicular to the growth direction, leading to the repetitive alignments of the lamellae. By using a moving window technique [36,53] a consistent growth of the solids is enabled, with a constant growth velocity and a constant solidification front temperature. This technique enables reduction in the effective domain size by shifting out the solidified phases from the simulation domain (see Figure 2 of [36]) and by subsequently applying an appropriate domain of the liquid phase at the opposite site. With this an infinite domain can be calculated in a fixed simulation domain. In simulation studies of systems in which solid/solid interactions can be neglected (such as in the current case with $D_{\hat{\alpha }S}=0$), this technique can reduce the computational effort and increase the accuracy considerably. In *DS* simulations, the interfacial energies are assumed to be isotropic ($\zeta_{\hat{\alpha }\hat{\beta }}=0$ in Equation (4)) and the temperature profile is set as Equation (5). Consequently, the investigated solidification velocities and the temperature gradient are taken from the experimental work of Witusiewicz et al. [30]. The authors of this reference indicate the microstructure, grown with a solidification velocity of v=0.5 µm s${}^{-1}$ and a constant temperature gradient of G=8 K mm${}^{-1}$, as the most perfect regular coupled growth with lamellar structure. For this condition, an average lamellar spacing of 23.4 µm is reported. Hence, in the following *DS* simulation studies, the microstructure evolution during the directional solidification is investigated for the growth velocity $v_{2}=0.5$ µm s${}^{-1}$ and two further velocities: $v_{1}=0.394$ µm s${}^{-1}$ and $v_{3}=0.678$ µm s${}^{-1}$. All three velocities are in the subsequently investigated velocity range of the *RDS* simulations. For each velocity, a two-dimensional simulation study with differing simulation domain widths is performed. Hereby, the domain widths for $v_{1}$ are varied from 20 µm to 36 µm, the domain widths for $v_{2}$ are varied from 15 µm to 35 µm and the domain widths for $v_{3}$ are varied from 14 µm to 30 µm. The simulations are performed for up to 48 h to calculate 35 million time steps, by using 20 cpus.

**Figure 3:**
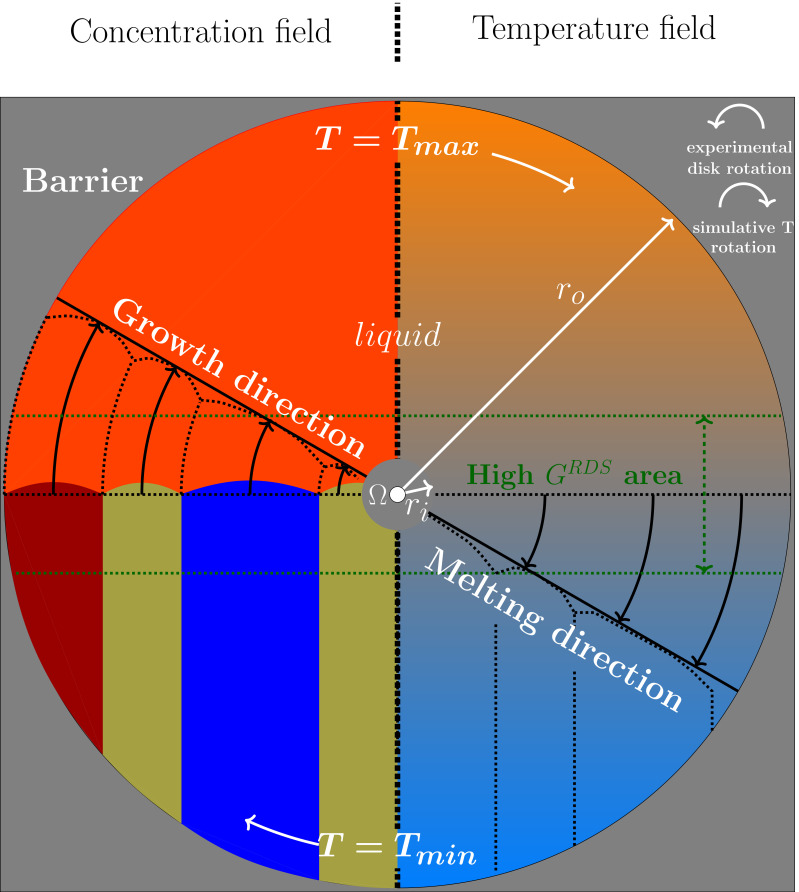
Schematic illustration of the rotating directional solidification simulation setup. The semicircle illustrates the concurrent influence of the concentration and temperature fields on the total domain.

Next, the setup for the *RDS* simulations is schematically introduced. The concentration field for the initial step of these simulations is schematically illustrated in the left half of Figure 3. In the right half of the image, the initial temperature field is shown. These half-domain illustrations are only used for the sake of brevity, whereas both fields affect the whole domain simultaneously. As the used multi-physics phase-field-framework Pace3D normally deals with rectangular simulation domains, a barrier section, similar to the works of [54,55,56], is introduced to create a circular simulation domain. In such a barrier region, neither the evolution equations (Equations (1) and (2)) nor the temperature profile (Equation (8)) are calculated. Due to the curved barrier region, the approximation of the gradient normals to the boundary can not be assumed as a linear problem, such as in [54,55,56]. At the boundaries of the curved barrier regions numerical instabilities can occur as a result of the contacting phase field. To omit these instabilities, a one cell thick layer of one of solid phases can be located permanently around the barrier. Hence, the barrier will always be in contact with a stable and converged phase field. Phase β, as an exemplary solid phase, is selected for this purpose, as it has the highest phase fraction of all solidifying phases. Due to this, a stability in the barrier boundary is expected. It has to be mentioned that, although this solid phase placement solves the mentioned problem in the barrier boundaries, it can affect the resultant phase fractions, as well as the lamellar spacings of the solidified phases. Considering the purpose of current work in *RDS* simulations which is a qualitative comparison of the obtained results with the available experimental data rather than reproducing them quantitatively, this effect is neglected by not considering near-barrier lamellae. With this, the impact of the boundary conditions on the finally drawn conclusions of the investigations is minimized.

At the middle of the domain melting and solidification conditions are located next to each other, which leads to a growth velocity of zero in the rotation center. In the experiments, this results in the formation of a single-phase area around the rotation center [22,57,58]. Due to the limited available computational resources a reduced number of evolving lamellae is investigated in a smaller domain compared to the experiments. Hence, such a single-phase region can lead to an instability of the growth conditions in the simulations as it affects the overall equilibria of the phase fractions in the domain. To avoid such unwanted effects on the growth of the neighboring solid phase arrangements, a second circular barrier section is introduced in the middle of the simulation domain. This barrier section is also surrounded by a one cell thick layer of the β phase.

The right hand side of Figure 3 shows the initial temperature field in a similar color labeling as Figure 1b. The region with a high temperature gradient is indicated by the green dotted lines. The area between these lines includes the solidification, as well as the melting front. Size of the used original square domain is 965×965 cells (301.8×301.8 µm). Bottom part of the domain with the lower temperature $T_{min}$, contains an initial amount of 13 sets of the desired αβαδ arrangement, all with a lamellar spacing of 23.4 µm. Due to the selected proportion of the inner and other radii of $\frac{r_{o}}{r_{i}}≃4.55$, which is similar to the radii proportions in Figure 5 of [22], five of the initial αβαδ lamellar sets build the growth front and the melting front of the simulation, respectively. The remaining three lamellar sets are placed beneath the barrier section around the rotation center. The upper half of the domain with the higher temperature $T_{max}$ is filled with the liquid phase. In this formulation, the direction of the temperature profile rotation is in opposite of the disk rotation in experimental works.

By using an angular velocity of ω=0.309 °s${}^{-1}$, the investigated velocities $v_{1}$ to $v_{3}$, from the *DS* simulations, adjust toward the centers of the three middle lamellae sets in the growth front, as well as in the melting front. Two different *RDS* simulations are performed in the following using a total number of 192 cpus, respectively. The first simulation is conducted with isotropic and the second with anisotropic interfacial energies. In the second case, all interfacial energies between the solid phases are determined to be anisotropic with equal strength. Both simulations are performed for two full rotations of 4π rad. The second rotations of 2π rad are carried out to check the stability of the system in formation of the solidification and the melting fronts, as well as the correct establishment of the obtained circular trajectories. Further, the convergence of the resultant lamellar spacings in the solidified phases is tested within the second rotation. The simulation of a complete 2π rad rotation demands an approximate calculation time of four weeks.

The system and process parameters used for the *DS* and for the *RDS* simulations are summarized in Table A3 of Appendix A. In the next section, the achieved simulation results are reported and discussed.

### 3.3. Directional Solidification (DS) Results

**Figure 4:**
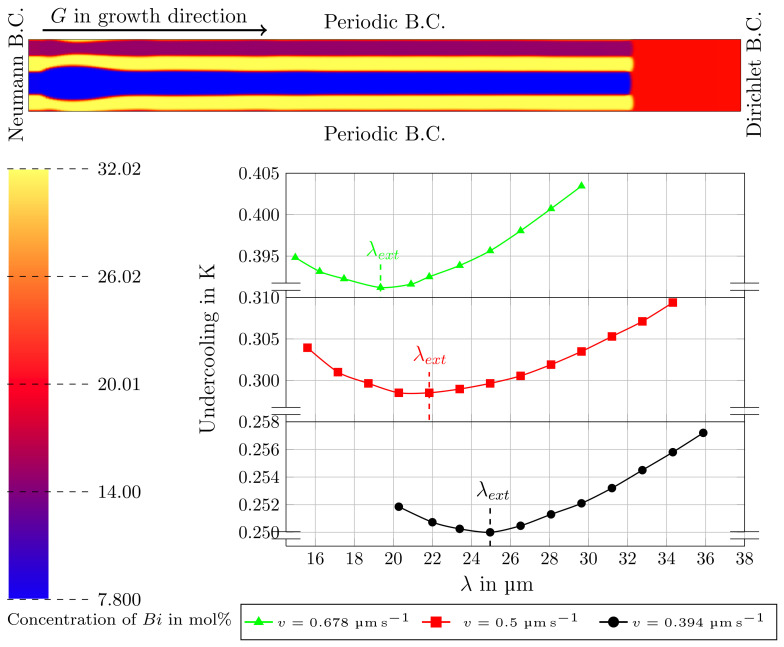
Top: Phase-field simulation result of the directional solidification in the Bi-In-Sn ternary eutectic system, with a solidification velocity of 0.5 µm s${}^{-1}$ and an applied temperature gradient of 8 K mm${}^{-1}$. Yellow: $\alpha =BiIn_{2}$, blue: β=β−In, violet: δ=γ−Sn, and red: liquid phases, respectively. Plot: Jackson–Hunt curve of the achieved undercoolings in different spacings.

The obtained simulation results for the directional solidification cases are illustrated in Figure 4. The top segment of the figure shows the stable growth of three solid phases into the liquid phase with an αβαδ stacking sequence. The domain size for this simulation is 75×150 cells equal to 23.4×46.8 µm. Using a moving window technique results in a final domain size of 23.4×237.12 µm. The evolving microstructure includes initial oscillations in the solid phase boundaries. These oscillations refer to the beginning of the simulation, where the curved interfaces between solid and liquid are established. The color bar shows the concentration of Bi in the phases involved, indicating a good agreement with the Calphad data of Figure 2 for these phases. Additionally, the diagram in Figure 4 depicts the Jackson–Hunt curves of the simulated system for the velocities $v_{1}$ to $v_{3}$. Each curve is constructed on the basis of the measured amounts of the resultant undercoolings at the different lamellar spacings. The general shapes of the curves clearly show a minimum undercooling-spacing points at $\lambda_{ext}$ and correlate well with the velocity dependent behavior expected from the Jackson–Hunt theory, which has also been observed in simulation studies of other material systems [33,49], in the literature. For each solidification velocity, the spacing at which the individual minimum of each curves occurs is labeled with $\lambda_{ext}$. The achieved amounts of $\lambda_{ext}$ for $v_{1},\;v_{2}$ and $v_{3}$ are equal to 24.96 µm, 21.84 µm and 19.34 µm, respectively. As it will be shown later, a good accordance between these amounts and experimentally reported data exists.

Instantly, based on the achieved theoretic validation of the modeled and simulated system in *DS* studies, the investigation of the rotating directional solidification simulation are performed in the following section. The results of the *DS* and *RDS* simulations are subsequently compared with the experimental predictions reported in [22,28,30].

### 3.4. Rotating Directional Solidification (RDS) Results

**Figure 5:**
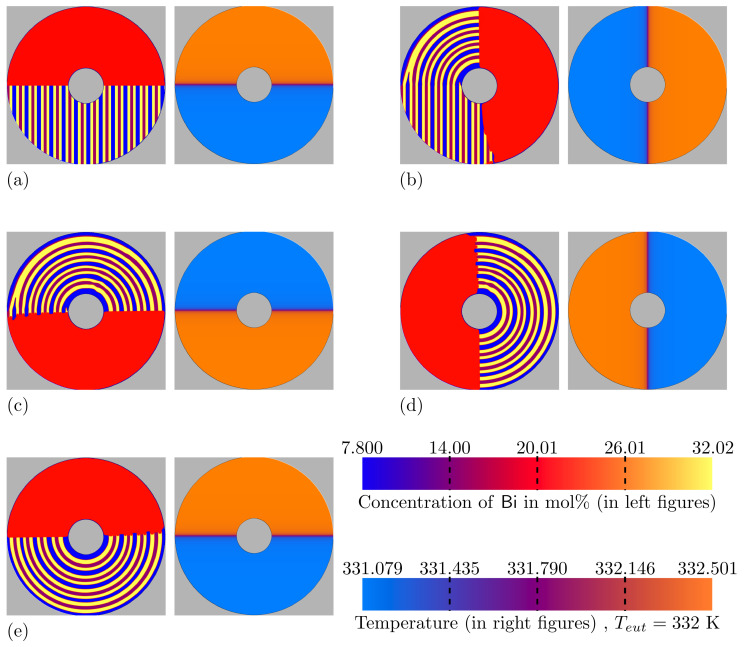
Simulation results for the isotropic system at different times, with a clockwise growth direction and a time step width of dt (see Table A3). (**a**) t=0, (**b**) t=24.2e6·dt, (**c**) t=48.4e6·dt, (**d**) t=72.6e6·dt, and (**e**) t=96.8e6·dt. At any stage, the left figure shows the phase fields (concentration of Bi) and the right figure shows the temperature field. Simulation domain size: 301.8×301.8 µm, $r_{o}=150.9$ µm, $r_{i}=33.15$ µm, ω=0.309 °s${}^{-1}$.

Figure 5 shows the obtained *RDS* simulation at different growth times for the isotropic simulation. In Figure 5a, on the left, the initial filling of the phases at t=0 is visualized by the concentration field of Bi, using the same color labeling as for the *DS* simulations in Figure 4. Due to the upcoming growth and melting events, the initial filling in the domain will melt as a result of temperature rise in the direction of the temperature field rotation, except for the growing solid/liquid interface. For the sake of simplicity, straight lamellae are filled, rather than the circular trajectories. In Figure 5a, on the right, the temperature distribution is depicted as formulated in Equation (8). In the simulation domain, two segments with almost unified colors of orange and blue can be observed, where one is at the top and one at the bottom of the simulation domain. Between these segments, there is a small area with a strong color transition. The uniform colors in both segments describe a low temperature gradient of 0.786 K mm${}^{-1}$, while the color transition describes a multiple times larger gradient, with a maximum of 240 K mm${}^{-1}$, in its center. Apart from the rotated temperature profiles, the other sub-figures of Figure 5 show the evolved microstructures at different simulation times. Good accordances of the solidification and melting front positions with the rotation of the temperature profile is achieved. Furthermore, similar to the *DS* simulations, the obtained concentrations for the elements Bi and In, in the phases observed at all times, are in good agreement with the plotted Calphad data of Figure 2. In Figure 5c, oscillations of the solidified phase boundaries can be observed after a rotation angle of approximately 120°. Similar to the *DS* simulations, the oscillations in the solid phase boundaries denote the transition region to the steady state condition. However, this effect occur after a certain solidification time, and not in the initial time steps, as is the case in the *DS* simulations.

**Figure 6:**
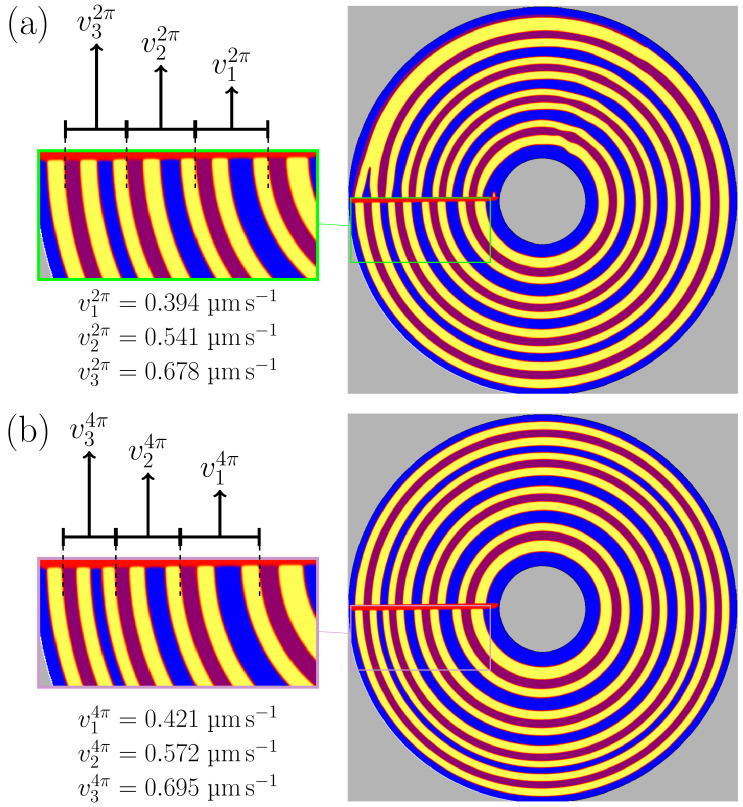
(**a**) Combination of the results of all simulation times for isotropic system in Figure 5, forming the 2π rad rotation image and the zoomed area of the evolved lamellae, with $v_{1,2,3}^{2\pi }$ growth velocities in their center. (**b**) The same configuration, at a rotation of 4π rad.

In Figure 6a, the results of the different time steps are summed up in one frame, showing the circular trajectories of the phase growth for one full rotation. The central three of the five initially filled lamellae are still intact with the stacking sequence αβαδ. In their center, these three lamellae sequences solidify with the growth velocities of $v_{1}^{2\pi }=0.394$ µm s${}^{-1}$, $v_{2}^{2\pi }=0.541$ µm s${}^{-1}$, $v_{3}^{2\pi }=0.678$ µm s${}^{-1}$. The directional solidification process of the lamellae sequences with similar velocities has previously been investigated in Section 3.3. Depending on these velocities, the lamellar spacings of the central three lamellae stackings are adjusting subsequently. These adjustments result in a rearrangement of the microstructure, which leads in the elimination of some phases next to the inner and outer boundaries. Similar effects can also be seen in the boundary regions of the experimental results from [21,22]. Due to these eliminations the initially set stacking of αβαδ is disturbed until the later elimination of the δ phase at a growth of approximately 120°. This elimination leads to the previously observed oscillations in Figure 5c. Due to the growth of the thin β phase layer at barrier boundary, a stable growth in the expected αβαδ stacking is established. The growth of the β phases at the boundaries, on one hand, is balancing the adjustments of the central lamellar stackings, but on the other hand this growth could also lead to the initial elimination of some solid phases next to the inner and outer boundaries.

In the sub-figure Figure 6b, the result after a rotation of 4π rad is shown. For this rotation, a stable continuous growth of the evolving lamellae can be observed, without any additional oscillations in the solid phase boundaries or any other unexpected phenomena. As the rotating centers of the disk (Ω in Figure 1 and Figure 3) and of the temperature field coincide, the desired circular trajectory for the growth of the solid phases results in the simulations. This agreement has not been reached in the mentioned experimental work of Mohagheghi et al. [22]. Due to the misalignment of the rotating centers, spiral growth trajectories of the solidified phases occur in the experimental micrographs, respectively. Furthermore, as the interfacial energies of the evolving phases are set isotropically in this simulation, no tilted growth with respect to the thermal gradient direction is observed. This observation is in agreement with evolution of the floating grains in the experiments [21,22]. The resultant growth velocities in the centers of the evolved lamellae are also shown in Figure 6, leading to the discrepancies of ${|}_{v_{1}^{2\pi }}^{v_{1}^{4\pi }}=6.5\%$, ${|}_{v_{2}^{2\pi }}^{v_{2}^{4\pi }}=5.7\%$, ${|}_{v_{3}^{2\pi }}^{v_{3}^{4\pi }}=2.5\%$.

**Figure 7:**
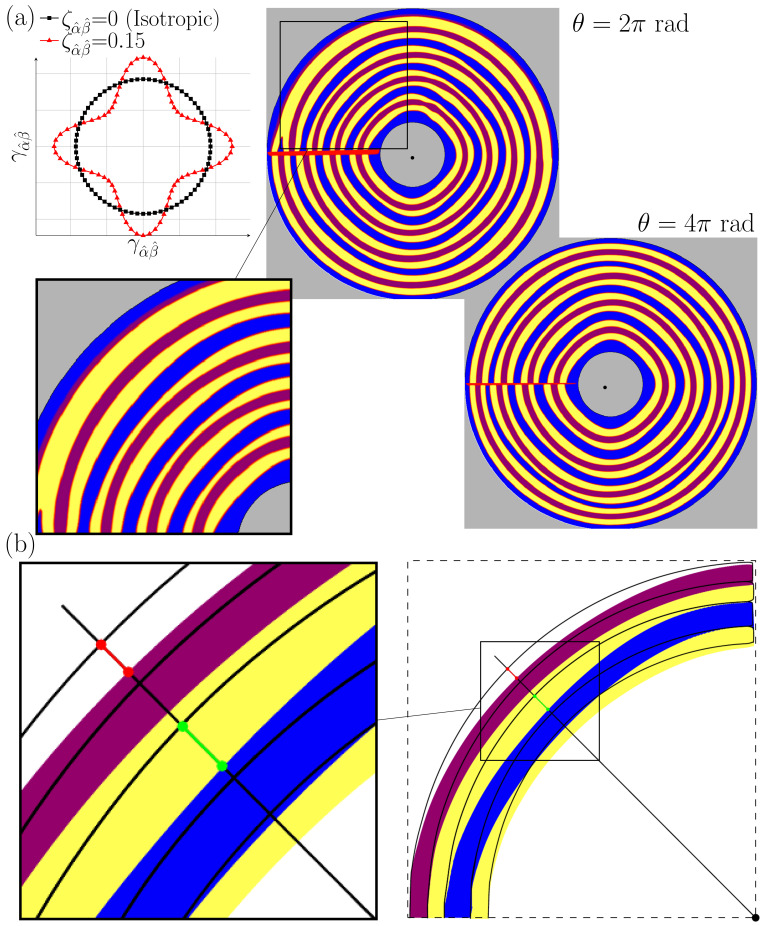
Effects of anisotropy in the interfacial energies on pattern formation. (**a**) The Wulff plot for anisotropy amount of 0.15 as well as the obtained microstructure at θ=2π and θ=4π rotations. (**b**) Comparison between the anisotropic simulation (colored phases) and the isotropic simulation of Figure 6 (black solid lines). Length of green line = 5.56 µm whereas red line length = 3.83 µm showing dominance of $\zeta_{\alpha \beta }$ in tilting phenomenon.

After the successful validating of the new simulation setup for *RDS* with isotropic material parameters, the influence of anisotropic interfacial energies is presented in Figure 7 after 2π rad and 4π rad rotations. The strength of anisotropy (ζ in Equation (4)) is set to an amount of 0.15 for all interfaces as illustrated in the Wulff plot of Figure 7a. The correlation of the lamellae with respect to the applied thermal gradient is achieved in accordance to the experimental works (see Figure 9 of [21] and Table 3 of [22]). For a more precise view on the anisotropy effects, the evolution of the lamellae after π/4 rad rotation is highlighted by an enlargement. As it can be seen, in the locked grains, the tilting is more pronounced in the αβ interfaces (yellow to blue) rather than the αδ interfaces (yellow to violet). In addition, Figure 7b shows the deviation of these anisotropic phase boundaries (colored phases) from the solid black lines denoting the isotropic simulation of Figure 6a for the middle lamellar stacking. In this sub-figure, the green line depicts the transition of the outer αβ phase boundary towards the rotation center at a rotational angle of π/4, whereas the red line delineates the same for the αδ phase boundary. The proportion of these two results has an amount of 1.45 and indicates more sensitivity of the αβ interface to the applied anisotropy compared to the αδ interface. This observation is in accordance with visual perception of [22], in which αβ interfaces are the main responsible for the formation of the crystallographically-locked grains. Similar to the isotropic simulation in Figure 6, the simulation with anisotropic interfacial energies show the elimination of some lamellae at the beginning of the first rotation and an oscillation of the solid phase boundaries after a rotation of approximately 120°. During the second rotation, a stable continuous growth of the evolving lamellae is observed either, without any additional disturbances of the growth morphology.

**Figure 8:**
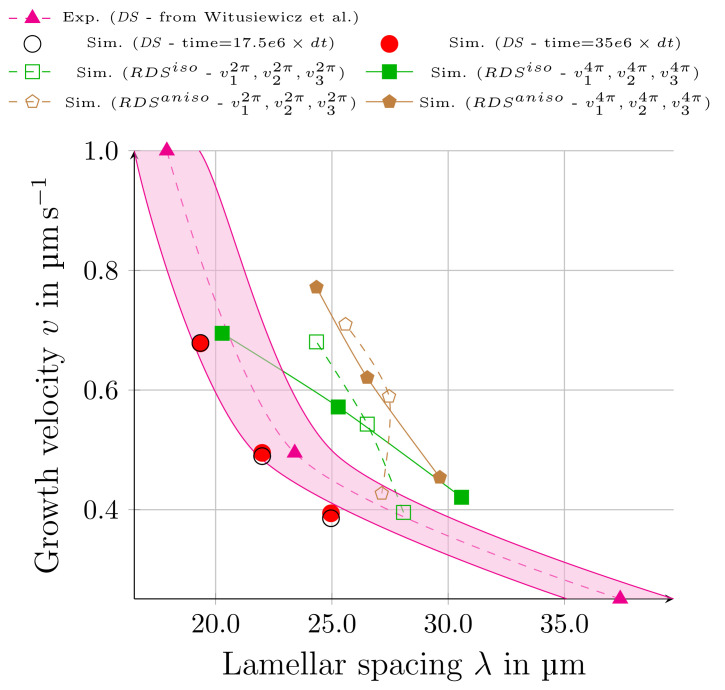
Comparison of the obtained Jackson–Hunt lamellar spacings for different solidification velocities in *DS* simulations (Figure 4), with the experimental results of Witusiewicz et al. [30]. $v_{i}^{n\pi }$ indicate the amounts for *RDS* simulations after nπ rad rotations (see Figure 6), respectively. Superscript iso stands for the isotropic simulation, whereas, aniso denotes the simulation with incorporation of anisotropy.

In Figure 8, the resultant amounts of $\lambda_{ext}$, in the *DS* and *RDS* simulations, and the experimental measurements of *DS* data in [30] are illustrated. The dashed magenta line is based on the average amounts of the achieved lamellar spacings for three different solidification velocities from *DS* experiments [30]. These data are depicted with solid triangles, and the highlighted surrounding area covers the experimentally observed minimums and maximums of the resultant lamellar spacings. The black unfilled circles illustrate the resulting lamellar spacings $\lambda_{ext}$ of the *DS* simulations after a simulation time of 17.5e6×dt, and the solid red circles attain the results after the full simulation time of 35e6×dt. The correlation between the filled and unfilled circles shows the convergence of the *DS* simulations to the steady state. As can be seen, the results of these simulations are mainly located in the highlighted region of the experimental *DS* data. This indicates a good accordance between the simulations and experiments for directional solidification. The green squares represent the results of the rotating directional solidification simulation with isotropic interfacial energies, $RDS^{iso}$, after a rotation of 2π and 4π rad. Similarly, brown pentagons denote the simulation with anisotropic interfaces, $RDS^{aniso}$, at the same rotational angles. For the isotropic simulation, the expected Jackson–Hunt relationship between growth velocity *v* and evolving lamellar spacing λ is observed. This correlation is more pronounced after the second rotation. For the case of $RDS^{aniso}$, this theoretic relationship is only found after the second rotation. The lamellar stackings with the slowest growth velocity has a smaller spacing than the lamellar stacking with the middle velocity after the first rotation, which is in contrast to the theory of Jackson and Hunt. This shows the effect of the anisotropy on the adjustment of stable growth conditions. Due to the applied anisotropic interfacial energies, the establishment of stable growth conditions requires a larger growth distance. This can be observed during the second rotation of $RDS^{aniso}$, in which stable growth conditions are obtained.

As a general conclusion of Figure 8, the obtained spacings indicate the convergence of the simulations and the correlation with the reported experimental results in representation of the anticipated lamellar spacings for *DS* simulations and qualitative agreements for the *RDS* simulations.

## 4. Summary and Outlook

In this work, phase-field simulations of the rotating directional solidification are performed, which are mentioned in the literature for the first time. This enables a precise study of the most important affecting parameters of the pattern formation, such as the solidification velocity, which can be studied as a process parameter, or the anisotropy of the interfacial energies, which can be regarded as a system parameter. In order to simulate this process, the effect of a rotating sample on the microstructure evolution, caused during the solidification, is rebuilt by an imprinted rotating temperature field. The modeled Bi-In-Sn system is validated in two-dimensional simulations of the *DS* process and is subsequently used to perform the simulation of the *RDS* process. By using isotropic interfacial energies, circular trajectories with the expected αβαδ repeat units are obtained, which represent the expected microstructure of the floating grains in *RDS* simulations. Furthermore, by introducing anisotropy of the interfacial energies into the material system, the growth of tilted lamellae is observed. In this case, the experimentally reported dominance of the αβ interfaces in the formation of the locked grains is recovered in the phase-field simulations. It has to be mentioned that, the effect of the applied thin β phase layer on the resulting microstructures can not be ascertained in its full extent. As the adjustment of the solid phases is influenced by several parameters, such as the growth velocities, the surrounding phases and the boundary conditions, an independent investigation of the different influences is required in forthcoming studies. For this, the implementation of a new barrier formulation without the need of an additional thin solid phase layer is planned, to improve the calculations of the interactions between the phase fields, concentration fields and a curved barrier section for upcoming investigations in future works. However, the presented results in this work show the general applicability of the presented method to investigate different growth morphologies within *RDS* simulations, which lays the foundation for further investigations. The effect of angular velocity variations on the microstructure evolution, for example, can be studied within the presented simulation setup. Initial simulations have already shown that an increase in the angular velocity ω can lead to instabilities, such as oscillations of the phase boundaries and the elimination of lamellae. Such behavior has also been observed in experimental works [23,28]. In addition, the systematic variation of anisotropic interfacial energies strength, is a further possible topic for upcoming research, which can be investigated with the presented *RDS* setup. The presented simulation setup is suitable for any eutectic material system which has 2D pattern in its stable growth, independent from the number of evolving phases.

## Figures and Tables

**Table 1:** Equilibrium concentrations of the involved solid phases in Bi-In-Sn system with eutectic composition at T=331 K, as an exemplary temperature, based on Witusiewicz et al. [29]. The concentrations of the liquid phase represent the concentration of the non-variant reaction at the eutectic temperature $T_{\mathrm{eut}.}$.

Phase	$c_{\mathrm{Bi}}$ in mol-%	$c_{\mathrm{In}}$ in mol-%	$c_{\mathrm{Sn}}$ in mol-%
γ−Sn	14.84	39.21	45.95
$BiIn_{2}$	32.01	66.67	1.32
β−In	8.02	66.96	25.02
liquid	20.37	60.36	19.27

## Data Availability

Not applicable.

## Abbreviations

The following abbreviations are used in this manuscript:

*DS*	Directional Solidification
*RDS*	Rotating Directional Solidification
Pace3D	Parallel Algorithms for Crystal Evolution in 3D

## Appendix A

**Table A1:** The approximated Gibbs energy functions for the Bi-In-Sn eutectic system, used for the phase-field simulations in this work, based on the introduced method of [33].

Parameters for the Gibbs Energy Function		
	$BiIn_{2}$	β−In
$a_{0}\left(T\right)$	3014.607T−896,340.473	−143.363T+55,902.219
$a_{1}\left(T\right)$	7087.078T−2,189,377.1803	138.230T−37,075.096
$a_{2}\left(T\right)$	3296.242T−929,782.259	355.122T−115,506.027
$a_{3}\left(T\right)$	−4116.795T+1,186,641.527	−255.378T+78,337.588
$a_{4}\left(T\right)$	−10,523.232T+3,220,675.0386	−198.487T+51,646.124
$a_{5}\left(T\right)$	4106.939T−1,263,269.831	3.884T−15,477.151
	γ−Sn	liquid
$a_{0}\left(T\right)$	−871.762T+301,469.411	−13.421T+13,192.535
$a_{1}\left(T\right)$	122.777T−26,128.366	12.381T+4229.506
$a_{2}\left(T\right)$	348.878T−139,478.362	23.675T−5488.634
$a_{3}\left(T\right)$	22.402T−5281.841	−20.932T−1575.961
$a_{4}\left(T\right)$	−154.236T+40,925.907	−14.338T−8078.906
$a_{5}\left(T\right)$	−30.836T−6902.264	−70.776T+8898.941

**Table A2:** 331 K is an exemplary temperature below the eutectic point, which is used to calculate and compare the amounts of the Gibbs energies *g* and the chemical potentials μ for the phases involved. cal: Calphad, app: approximated, eq: equilibrium.

Comparison of Calculated Values from Gibbs Energy Functions				
and the Calphad Database in J mol${}^{-1}$.				
	$BiIn_{2}$	β−In	γ−Sn	liquid
$g_{\mathrm{cal}}^{eq}{|}_{331K}$	−20,885.512	−20,047.493	−19,650.776	−20,315.829
$g_{\mathrm{app}}^{eq}{|}_{331K}$	−20,879.5	−20,042.9	−19,632.7	−20,315.536
$\mu_{\mathrm{app},0}^{eq}=\frac{\partial g_{\mathrm{app}}}{\partial c_{\mathrm{Bi}}}{|}_{331K}$	−3522.53	−3470.85	−3449.36	−3521.90
$\mu_{\mathrm{app},1}^{eq}=\frac{\partial g_{\mathrm{app}}}{\partial c_{\mathrm{In}}}{|}_{331K}$	−2294.85	−2266.95	−2315.73	−2294.48

**Table A3:** Summary of the dimensionless simulation and numerical parameters, as well as their amounts in physical units, for simulation studies of the Bi-In-Sn system—$\alpha :BiIn_{2}$, β:β−In, δ:γ−Sn, L:liquid, S:Solid, iso: isotropic simulation, aniso:: anisotropic simulation.

Parameter	Simulation Value	Physical Value	
$\gamma_{\alpha \beta }$,$\gamma_{\beta \delta }$,$\gamma_{\beta L}$	$9.26\cdot 10^{-4}$	0.2888 J m${}^{-2}$	
$\gamma_{\alpha \delta }$,$\gamma_{\alpha L}$	$2.78\cdot 10^{-4}$	0.0866 J m${}^{-2}$	
$\gamma_{\delta L}$,	$4.63\cdot 10^{-4}$	0.1444 J m${}^{-2}$	[59]
$\gamma_{\hat{\alpha }\hat{\beta }\hat{\delta }}$	15·γ	-	
$A_{0}$	0.001374	0.456 K	
$T_{m}$	0.99937	331.79 K	
$T_{eut.}$	1	332 K	[29]
$D_{\hat{\alpha }L}$	0.62	$10^{-9}$ m${}^{2}$ s${}^{1}$	[60]
$D_{\hat{\alpha }S}$	0	0 m${}^{2}$ s${}^{1}$	
$G^{\mathrm{DS}}$	$7.52\cdot 10^{-6}$	8 K mm${}^{-1}$	
$G^{\mathrm{RDS}}$	$7.4\;\cdot 10^{-6}-2.255\;\cdot 10^{-4}$	0.786–240 K mm${}^{-1}$	
ω	$3.24\cdot 10^{-7}$	0.309 °s${}^{-1}$	
$T_{eut}$	1	332 K	
dx	1.0	0.312 µm	
dt	0.2	$1.2\cdot 10^{-5}$ s	
$\tau_{\alpha L}$,$\tau_{L\alpha }$	1.45		
$\tau_{\beta L}$,$\tau_{L\beta }$	2.73		
$\tau_{\delta L}$,$\tau_{L\delta }$	1.31		
$\zeta_{\hat{\alpha }\hat{\beta }}^{iso}$	0	-	
$\zeta_{\hat{\alpha }\hat{\beta }}^{aniso}$	0.15	-	

**Table A4:** Thermo-physical properties of Bi-In-Sn system at melting point from [61].

Property	Amount	Unit
Thermal expansion coefficient	6.11	°C${}^{-1}$
Average density	8.81	g/cm${}^{3}$
